# A Multidimensional Approach to Measuring Economic Insecurity: The Case of Chile

**DOI:** 10.1007/s11205-022-02918-5

**Published:** 2022-04-11

**Authors:** Joaquín Prieto

**Affiliations:** International Inequalities Institute, London School of Economics & Political Science (LSE), Houghton Street, London, WC2A 2AE UK

**Keywords:** Economic insecurity, Global South, Multidimensional index, Survey of households finances, Well-being, D63, I31

## Abstract

This paper proposes a strategy to measure economic insecurity in countries in the Global South. It builds a 'Multidimensional Economic Insecurity Index' (MEII) that combines four indicators of economic vulnerability that cause stress and anxiety: unexpected economic shocks, unprotected employment or non-workers in the household, over-indebtedness and asset poverty. The index offers a measure that directly relates economic uncertainty to stress and anxiety due to the lack of protection and buffers to face an unexpected economic shock. The MEII is applied to Chile using Survey of Household Finances (SHF) cross-sectional data (2007, 2011, 2014 and 2017). The results show that (i) about half of the Chilean households experienced, on average, two or more economic vulnerabilities during the last decade with an intensity of 2.3 vulnerabilities, and (ii) economic insecurity affects households on the entire income distribution, even in the highest income deciles groups. By identifying the groups of households most affected by economic insecurity and its trend in recent years, applying the MEII in countries such as Chile provides relevant information to monitor, evaluate and improve social safety nets besides labour market regulations.

## Introduction

The design of better policies that could lead Latin American countries towards more inclusive and sustainable development requires new measures of progress and well-being (OECD, CAF, ECLAC, & EU, 2019). Well-being measurements are embedded in the 17 Sustainable Development Goals (SDGs) 2030 agreed by all UN member states. However, the only SDG goal that actually mentions well-being is SDG 3, "Good health and well-being", yet, the indices used are insufficient to measure well-being since the economic risk is not considered. This is a crucial aspect to consider knowing the vast impact of the last two global crises -the Great Recession 2007–2008 and COVID-19 pandemic- on the Latin American population's well-being.

In the context of high levels of income inequality combined with weak social security systems, Latin American countries have failed to protect most households against economic shocks. Despite the efforts of governments to support the most vulnerable families, a high proportion of households have experienced unemployment, descending income mobility, and sharp falls in their assets (wealth), all of which have contributed to an increase in economic insecurity (Hacker, 2019; Rohde & Tang, 2018).

In (2009), Stiglitz, Sen, and Fitoussi highlighted the importance of measuring economic insecurity to understand how economic risks are related to individuals' well-being and designing social policies based on a broader perspective than the one obtained through static measures of poverty and material deprivation. Since then, several authors have proposed measures of economic insecurity that address the stress and anxiety produced by exposure to adverse economic events and the incapacity to face them when they occur.^1^ This paper proposes a multidimensional measure of economic insecurity at the household level that is applicable to contexts where: (i) inequalities in household wealth are high, (ii) the social safety net is limited, (iii) indebted households are increasing due to strong credit growth, and (iv) the reduction of absolute income poverty rather than relative poverty is the primary policy concern.

In upper-middle-income economies such as Chile, Brazil, Colombia and Mexico, there is little theoretical or empirical debate around economic insecurity, even though a large proportion of the population is exposed to economic shocks that not only generate income losses for the households but also lead them to experience poverty. In the Latin American region, the social group most exposed to economic shocks has been described as the 'strugglers' (Birdsall et al., 2014) due to the permanent effort made by this type of household to maintain their income levels. This social group faces high economic insecurity since they have neither sufficient assets to offset an economic shock nor access to unemployment insurance or compensation in case of dismissal when working in the informal sector.

The emergence of this group of households that are vulnerable to poverty in Latin America has been accompanied by a massive increase in access to credit for consumption and mortgages (Matos, 2017), which is presented as a credit boom instead of a financial deepening (Hansen & Sulla, 2013). This rapid growth of credit access increases the risk of over-indebtedness in low-income households (Guérin et al., 2013; Schicks, 2013). In addition, several countries in Latin America are highly vulnerable to natural disasters such as floods, droughts, and earthquakes, which cause aggregate shocks to both the assets and income of households living in the affected areas.

In this context, the adverse effect on households' well-being generated by the uncertainty of not being able to cope financially with an unexpected event that triggers an economic loss is studied. To do so, I build four objective indicators (unexpected economic shocks, unprotected employment or non-workers, over-indebtedness, and asset poverty) for two dimensions of economic insecurity: (i) household risk to an unexpected economic event, and (ii) lack of household buffers to face an economic shock. I combine these indicators using a multidimensional approach to build an adjusted multidimensional vulnerability rate called the 'Multidimensional Economic Insecurity Index' (MEII). This approach has two stages. First, I identify the economic vulnerabilities, and then, I apply an aggregation procedure to integrate the multidimensional information on economic insecurity into a single scalar measure (Alkire & Foster, 2011). The Chilean Survey of Household Finances (SHF) cross-sectional data (2007, 2011, 2014 and 2017) are used to build the MEII.

The MEII has two advantages that make it an appropriate measure for policy analysis. The first advantage is that it simultaneously measures the incidence (proportion of economically insecure households) and the intensity of the economic insecurity (number of vulnerabilities affecting it). The second advantage is that the MEII can be decomposed by population subgroups (e.g., income decile groups or geographic areas) and economic insecurity domains (e.g., employment, income, indebtedness, and wealth). Thus, it allows for monitoring each of the dimensions of insecurity that are targeted by multi-sectoral policy strategies such as unemployment insurance, investment in social and affordable housing, micro-finance interventions, cash transfers, and policies to stimulate saving, among others. It is the first time to apply the concept of economic insecurity to middle-income countries and complement other well-being measures, such as vulnerability to poverty and multidimensional poverty, which are more commonly used in these countries.

The estimates for Chile between 2007/2017 show high levels of economic insecurity in regard to both the risk of an unexpected economic event and the lack of a household buffer to offset a potential loss. More than a third of households were exposed to unexpected economic shocks during this period. The indicators providing information about households’ lack of protection reveal that 62.8 per cent were asset poor, 30 per cent had only unprotected workers or non-workers, and 15.4 per cent faced over-indebtedness. Combining the measures in the MEII, we found that, on average, about half of Chilean households experienced two or more economic vulnerabilities during the last decade, with an intensity of 2.3 vulnerabilities. The index is correlated with more conventional macro indicators such as the GDP growth rate and labour informality rate. The highest index levels are between 2007 and 2011, followed by a significant decrease between 2011 and 2014, increasing again between 2014 and 2017.

This paper makes three contributions. First, from a conceptual point of view, it uses two dimensions of economic insecurity related to an unexpected economic event and the household buffer to protect from this potential economic loss. Although both dimensions (and their respective indicators) are sources of insecurity, each of which may trigger stress and anxiety in individuals and households, the origin of these adverse psychological effects differs. In previous work, the focus in terms of the selection of indicators has either been on choosing between subjective and objective indicators (Rohde et al., 2015; Romaguera de la Cruz, 2020) or on just one source of economic insecurity (Azzopardi, Fareed, Lenain, & Sutherland, 2019; Balestra & Tonkin, 2018; Bossert & D’Ambrosio, 2013; Hacker et al., 2014; Rohde et al., 2014).

Second, it proposes indicators for these two dimensions of economic insecurity to be implemented in middle-income countries, especially those in Latin America, delivering a measure of well-being that contemplates the possibility of future events, which complements the forward-looking measures of vulnerability to poverty used in these countries. Until now, all measures of economic insecurity at the household or individual level have been applied using data from developed countries. Applying the MEII to Chile for the period 2007–2017, economic insecurity is studied in a nation characterised by (i) a significant reduction in absolute poverty coupled with a significant increase in vulnerability to poverty (Prieto, 2019); (ii) an unemployment insurance system that has not yet managed to cover the workers who have greater job instability (Sehnbruch et al., 2018); (iii) an increase in consumer debt that has been accompanied by mental health problems in households facing over-indebtedness (Hojman et al., 2016); and (iv) a high proportion (75 per cent) of households experiencing asset-based poverty according to the OECD measure (Balestra & Tonkin, 2018), placing Chile, in this aspect, within the most economically vulnerable OECD countries.

Third, from a social policy perspective, the new measure of economic insecurity makes a significant contribution to measuring progress in the region. The MEEI index goes above and beyond the Gross Domestic Product (GDP) per capita or the Human Development Index (HDI) (Barcena, Manservisi, & Pezzini, 2017). It could be used as a design and monitoring tool for countries that are in the process of defining a new social contract based on a more robust social protection system centred on people’s well-being (OECD, 2020).

This paper is organised as follows. Section 2 summarises the most salient theoretical approaches and empirical findings related to economic insecurity. Section 3 describes the SHF data and dimensions and indicators of economic insecurity used in the research. Section 4 explains how the index of economic insecurity is built. Section 5 shows and discuss the empirical results. Section 6 presents the conclusions.

## Background

During the last decade, new approaches have been proposed to measure the social and economic well-being of the population (e.g., the *Equitable and Sustainable Well- being* (BES) project (Alaimo et al., 2020). These approaches go beyond gross domestic product (GDP) to measure welfare, acknowledging that production is not an appropriate indicator of individual and social well-being. One of the new well-being metrics that has been studied theoretically and empirically at both levels is economic insecurity (Hacker, 2018; Osberg, 2018; Rohde & Tang, 2018). The notion of economic insecurity refers to "the adverse well-being effect of (involuntary) exposure to uncertainty in enduring an uninsured financial shortfall" (Rohde & Tang, 2018, p. 303). The idea behind it is that economic insecurity has a subjective component and is a forward-looking measure since stress and anxiety are associated with financial uncertainty. This measure assumes that changes in the subjective levels of anxiety in regard to lacking economic safety are highly correlated with changes in the objective risk (Osberg, 1998; Osberg & Sharpe, 2014).

### Economic Insecurity as a Measure of Well-Being

The importance of economic insecurity as a measure of well-being is recognised in the Human Development Report (HDR) by the United Nations Development Program (1994), which states that economic security "requires an assured basic income for individuals, usually from productive and remunerative work, as a last resort, from a publicly funded safety net" (HDR, p. 25). Beyond this formal recognition, the value of measuring economic insecurity is that provides estimates on two key welfare costs associated with it. First, economic insecurity makes difficult for households with children to plan for the future, resulting in psychological distress in the household environment and in diminished well-being, human capital investment, and development of the children in the household (Hardy, 2014; Hill et al., 2013; Western et al., 2016).

Second, economic insecurity can influence complex psychological processes that cause an increase in health problems throughout people’s lives (McEwen & Gianaros, 2010). Several studies have found that the physical and mental health of household members is affected by different downside risks of future economic events, such as sharp income drops or unemployment (Adda et al., 2009; Caroli & Godard, 2016; Ferrie et al., 2005; Kopasker et al., 2018; Smith, Stoddard, & Barnes, 2009).

Studies have shown that households that experience difficulties in raising emergency funds when facing an unexpected economic shock are associated with poor health outcomes (e.g. Rohde et al., 2016). More specifically, households lacking access to health insurance, and households financially fragile due to high indebtedness show higher prevalence of physical and mental health problems such as obesity, anxiety and depression (Clayton et al., 2015; McWilliams, 2009; Münster et al., 2009; Sweet et al., 2013, 2018).

A direct association between economic insecurity and subjective well-being has also been found, for example, the negative relationship between job insecurity and life satisfaction in countries such as Australia, Germany and the United Kingdom (Clark & Georgellis, 2013; Green, 2011; Otterbach & Sousa-Poza, 2016), and the positive correlation between the universal coverage of health insurance in one of the states in the U.S.A. and the levels of happiness of the affected population (Kim & Koh, 2018).

Although economic insecurity has serious implications for well-being, there is no commonly accepted framework for its analysis. This can be explained by the methodological challenges in its operationalisation. First, it is difficult to know whether the economic shocks experienced by a household are unexpected or the result of a household decision. Second, economic insecurity is a phenomenon that deals with unobservable and forward-looking expectations rather than retrospective information. Third, it is reasonable to think that two individuals with similar characteristics may have very different perceptions about the future. Hence, under the same conditions, one individual can feel much more insecure than the other.

As a consequence, the empirical studies that have been carried out in developed countries up to now have proposed their own definitions of economic insecurity along with an ad hoc methodology for their measurement (Hacker, 2018; Osberg, 2018; Rohde & Tang, 2018).

### Aggregate and Individual-Level Measures of Economic Insecurity^2^

When making comparisons across countries, the aggregated national indices allow for analysing trends in economic insecurity based on the combination of a variety of economic risk indicators. The two main macro indexes of economic insecurity are the Index of Economic Security (IES) proposed by Osberg and Sharpe (2014), and the International Labour Organization’s (ILO) index of economic security (ILO, 2004). The IES comes from the ‘named risks’ approach, which examines four downside economic risks named in Article 25 of the UN Universal Declaration of Human Rights (i.e., unemployment, family breakup, medical costs, and poverty in old age). Osberg and Sharpe (2014) applied an IES adjusted to 70 countries and found substantially different levels of economic insecurity across rich and developing countries. The ILO index uses aggregated data from countries to measure seven forms of labour security (income, labour market, employment, work, skills, job, and voice representation). This index is currently applied to 90 countries, covering 86 per cent of their population (Rohde & Tang, 2018).

Using a one-dimensional-micro-based measurement approach, economic insecurity has been conceived of as (i) job insecurity (Keim et al., 2014; Rehm, 2016), (ii) a large income loss experience or a downward deviation from trend income (Hacker et al., 2010, 2014; Rohde et al., 2014; Western et al., 2016), (iii) financial difficulties (over-indebtedness and arrears) (Anderloni et al., 2012; Azzopardi et al., 2019; Białowolski, 2018), and (iv) an inadequate private wealth buffer stock against shocks (Balestra & Tonkin, 2018; Bossert & D’Ambrosio, 2013). Most of these measures of economic insecurity have focused on the United States, showing a significant increase in recent decades, with peaks in the years 1998 and 2007. Nevertheless, integrated measures fail to include important dimensions of economic insecurity in their construction, focusing on large income loss or the buffering role of private wealth or income volatility (Osberg, 2018). For example, they fail to capture the social protection that the state can provide (e.g. eligibility for unemployment insurance benefits or severance payments) or do not incorporate subjective measures on the perception of the economic situation that reflect the anxiety and concern of individuals in a direct way (Espinosa et al., 2014).

In recent years authors have wondered whether economic insecurity is also increasing in other developed countries using a multidimensional index that combined all of the unidimensional measures into a single indicator (Rohde et al., 2015; Romaguera de la Cruz, 2020). The rationale behind this multidimensional measure is that an appropriate concept of economic insecurity should be able to capture all types of economic stress explained by the risk of a negative financial future. Rohde et al. (2015) analysed the case of Australia using indicators of job insecurity, financial dissatisfaction, emergency funds, unemployment risk, expenditure distress and income drop, and found that these are correlated with the country’s unemployment rate and GDP growth rate. Following a similar methodology, Romaguera de la Cruz (2020) apply a multidimensional measure of economic insecurity in three developed countries, being significantly present in middle-income households both in Spain and France but not in Sweden.^3^

It is worth mentioning that economic insecurity measures at individual-level have not been developed in the Global South.^4^ Although Osberg & Sharpe’s IES proposed a multidimensional measure at the aggregate level to compare both Global North and Global South countries, indices of economic insecurity at the individual or household level allow for comparative analysis between different groups within each country, making them a key tool for the design of social protection policies that can offer a better safety net to protect households from the stress or anxiety caused by not being economically prepared to face different economic shocks in the future.

## Data and Measures of Economic Insecurity

### SHF Data

I use data from the Chilean Survey of Household Finances (SHF) carried out by the Central Bank of Chile in 2007, 2011, 2014 and 2017. The SHF is representative of urban private households. It collected information on income, expenditure, household characteristics, household assets and debts with a high degree of detail.^5^ The SHF used a stratified, multi-stage probability sample selected from the population Census (2002 and 2012) sampling frame and included an oversample of well-off households using taxpayer information from the Chilean Internal Revenue Service (SII for its acronym in Spanish). The SHF design is similar to that of the U.S.A. Survey of Consumer Finances (Kennickell & Woodburn, 1999), as well as of the Household Finance and Consumption Survey coordinated by the European Central Bank (HFCN, 2016).^6^

I use the SHF household-level data, which not only contains variables on financial and non-financial assets and debts, but also include socioeconomic and demographic variables. The size of the sample in 2007 was 3827 households. The 2011 sample comprised 4057 households and the samples in 2014 and 2017 comprised 4502 and 4449 households respectively.

In summary, the economic insecurity variables obtained from the SHF are (i) employment status and type of contract of household members; (ii) retrospective questions related to significant unexpected expenses or substantial unexpected income drops faced by the households in the last 2 years; (iii) information on the burden that debt imposes on the income of household; and (iv) household assets such as non-housing wealth.

I use the Chilean national poverty line defined by the Ministry of Social Development (2015), which measures poverty in absolute terms. This threshold is based on the cost of a basic food bundle. I construct post-transfer household income as the sum of income from labour, imputed rent, and private transfers plus public transfers. Because I use assets as a stock of material resources that can support the current consumption of a household, it is appropriate to equivalise it in the same way as household income is equivalised (OECD, 2013, p. 141). Therefore, to account for different disposable income and asset requirements for different family sizes, I equivalise both income and assets using the scale that is equivalent to the household size to the power of 0.7.^7^

I break down the estimates of the Multidimensional Economic Insecurity Index by individual characteristics such as gender, age and education level and household characteristics such as family type (couple or single, with or without children, or lone person), size of the household, housing (outright owner or owner with mortgage or tenant) and location (regions).

### Measures of Economic Insecurity

To measure economic insecurity requires quantifying the level of stress or anxiety of a household attributed to an uncertain financial future. Given that stress or anxiety is not directly observable in the data sources that sociologists and economists usually work with, sources of economic insecurity rely on proxies. I classify these proxy measures into two dimensions. The first dimension is the risk of the household experiencing potential events related to negative economic consequences such as unemployment, losses in asset values, or unexpected medical expenses. The second dimension is the lack of household economic buffers, which generates stress such as not having enough assets to face an event that decreases incomes or increases expenses, or not having access to social protection mechanisms to offset these economic losses.

Following an approach focused on the household-level measures, due to the existence of a shared decision-making process, the index uses four sources of stress distributed across two dimensions of the economic insecurity. As mentioned above, the first dimension is vulnerability to economic loss. I consider in this dimension a measure of unexpected large income loss or unanticipated expenses (known as downside income insecurity). The lack of household buffers is the second dimension. It includes three measures: (i) unprotected employment or non-workers in the household, (ii) over-indebtedness, and (iii) asset poverty.

The starting point in the selection of these indicators is that the level of stress or anxiety of an individual or household depends on the combination of these four sources of economic insecurity. For example, a family facing a decrease in their income (e.g., losing their job without access to unemployment insurance) might spend their savings or borrow money. However, families that have low levels of savings, or that have a limited ability to borrow, or are already allocating a large portion of their income to servicing a debt, may have trouble addressing this unexpected drop in earnings and be forced to give up food or fail to pay their debts or other receipts.

I justify selecting the four sources of economic insecurity relying on the empirical evidence offered by the health economics literature. Several investigations have linked these economic vulnerabilities with health problems, in particular with the stress of the household’s members. These references are illustrated below in the description of the indicators for each source of economic insecurity.

#### Household Risk to An Unexpected Economic Event

Income insecurity refers to the risk of large income drops or unexpected expenses faced by families should they encounter unpredictable events of social life (Western et al., 2012). In addition to unemployment risk, the common triggers of income insecurity are family breakdown and illness. Concerning health problems, these not only cause losses in household income (e.g. independent or informal workers with no protection for this type of incidents) but also unanticipated costs whereby part of the household income has to be used for medical expenses (Adda et al., 2009). Studies have shown that household experiences of income instability are associated with situations of stress in parents and children, and increase the likelihood of indebtedness of the household, inconsistency in consumption, and underinvestment in children (Hill et al., 2013; Western et al., 2016; Yeung, Linver, & Brooks–Gunn, 2002).

The indicator to measure income insecurity is based on the following SHF retrospective question: “Have you faced either unexpected expenses of significant magnitude or an income drop of significant magnitude during the last year?”.^8^ Although it is not an objective measure such as a household disposable income fall, it does ensure that the economic shock is considered as unexpected and not a household decision. I change this dichotomous indicator for a measure of risk attached to each household making out-of-sample predictions. I use a probit model, in which the dependant variable takes the value 1 if the household faced any economic event that triggered a sharp drop in income or a sharp increase in their expenses in $t-1$, and 0 otherwise.^9^

Assuming the relation stays the same for the next period, I attached to each household the predicted probability calculated using the characteristics at the current period and coefficients from the regression of that year. I classify a household as income insecure if the risk of an unexpected economic event is situated above a threshold. I follow a data driven strategy to define the cut-off (Romaguera de la Cruz, 2017). I establish the 70th percentile of predicted probabilities as a threshold because it is the cut-off that is closest to the observed values after doing sensitivity analyses for different thresholds. In this way, I differ from authors who have measured a similar economic insecurity dimension (a drop in household income) in their multidimensional index following the proposal of Hacker et al. (2010).^10^

#### Lack of Buffers to Offset Potential Economic Loss

- Unprotected employment or non-workers in the household.

Although I do not include an indicator that would account for the risk that a household has of an economic shock due to a job loss, I do consider the current employment situation of the household members in regard to facing an event of this type in the future. The term informal employment is used to refer to a lack of economic protection in the case of dismissal or a work accident. Salaried workers that do not have health and social security contributions as part of their labour relationship with their employer have an informal occupation. Self-employed workers and employees who are part of the informal sector (that is, their businesses are not registered in the Internal Revenue Service) are informal workers (ILO, 2013).

The SHF collects information on the occupational category and the type of contract of all the members of the household that are working at the time the survey is applied. Also, it asks whether or not household members pay social security contributions. This information allows me to construct a variable that distinguishes an informal worker from a formal one. I start from the assumption that formal jobs normally offer economic protection in the case of dismissal.

For households without any labour market attachment, I consider that they are also unprotected against an unexpected economic shock. This consideration is important because these types of households could be classified as economically secure as they do not have informal workers. Thus, the objective indicator of economic insecurity for each household works as follows: I classify a household as economically insecure if (i) none of the workers in the household has access to unemployment insurance benefits or would receive any sort of compensation in the event of their dismissal, or (ii) the household does not have members working. This indicator takes the value of 1 when all workers are informal workers or there are non-workers in the household.

(b) Over-indebtedness.

There is abundant evidence on the effect of over-indebtedness and its associated financial difficulties (e.g., unsuitable debt or debt arrears), causing adverse psychological effects such as distress, anxiety, reduced life satisfaction and depression in the members of a household (Białowolski, 2018; Białowolski & Weziak-Bialowolska, 2014; Bridges & Disney, 2010; Brown et al., 2005; Hojman et al., 2016; Selenko & Batinic, 2011; Sweet et al., 2013). Although it is impossible to know for sure whether over-indebtedness is due to an unexpected event or a risky planned decision, a household with a high default risk experiences high stress due to its susceptibility to any future economic shock, even if this is not a significant loss.

The association between debt and adverse psychological effects seems to be driven by non-mortgage debt such as consumer credit (see Hojman et al., 2016). For this reason, the vulnerability indicator focuses on over-indebtedness of consumption. In the SFH, respondents report the household’s consumer debt amount (e.g., bank consumer loans, bank credit cards, and retailers’ credit cards) and its financial burden.^11^ I follow Disney et al. (2008) strategy to define when a household is over-indebted. These authors suggest that if a family spends more than a certain threshold of its total monthly income on unsecured debt repayments, it should be classified as over-indebted.^12^

The steps to identify if a household is over-indebted are two. First, I calculate the financial burden relative to the monthly household income. It corresponds to the ratio of the monthly repayment of unsecured debt to monthly household disposable income. Second, I use a 40 per cent threshold to define over-indebtedness. This cut-off is higher than the one suggested by Disney (25 per cent) but similar to the threshold used by Hojman et al. (2016). I used a statistical optimality criterion to obtain the latter. To sum up, households spending more than 40 per cent of their monthly disposable income on consumer debt repayments are considered over-indebted in this vulnerability indicator.

Although the SHF asks the interviewee about their perception of the household level of indebtedness, I use only the observed information about this source of economic insecurity. By doing so, I avoid introducing potential subjective bias related to the idiosyncratic characteristics of individuals in the construction of the indicator.

(c) Asset poverty.

A household that does not have an adequate buffer (wealth) against major economic shocks is aware of its economic vulnerability generating stress and anxiety among its members (Bossert & D’Ambrosio, 2013). The economic literature focused on the lower part of the income distribution has measured this economic disadvantage as asset poverty (Brandolini et al., 2010). A household is considered to experience asset poverty if its assets (e.g. non-housing wealth or liquid assets) are insufficient to keep it above the poverty line for a specific period of time (e.g. 3 or 6 months) (Haveman & Wolff, 2004).

The first step to build this vulnerability indicator is to set the wealth measure. The SFH collects information on household wealth, such as assets and liabilities, which allows using a non-housing wealth as a household asset measure. Then, I define the wealth measure as the difference between total assets and liabilities (without considering any wealth or debt related to the primary residence). Although several studies use liquid assets in the asset-poverty operationalisation (Balestra & Tonkin, 2018; Hacker et al., 2014), I decided not to use this in the case of Chile for two reasons. First, when applying the liquid assets measure to Chile, 9 out of 10 households fell into asset poverty in the 10 years analysed (2007/2017). Hence, this definition provides little information for middle-high income country contexts like the Chilean. Second, including tangible assets such as vehicles in the operationalisation of asset-poverty allows for considering, for instance, selling the car as a concrete and feasible strategy of the household to address an income shock in this type of national context.

The second step is to choose both a poverty line and the length of time where a household can live only with the liquidity of its assets without falling into poverty. I use the relative poverty line suggested by the OECD to compare the poverty rate among its countries members. It corresponds to the half of median household equivalised income of the total population. For the period threshold, I use 3 months. It is a less demanding cut-off than 6 months and is commonly used to measure asset poverty.^13^

In summary, for this vulnerability indicator, a household is asset poor if its non-housing wealth is less than the value of three poverty lines.

In total, I generate four measures of economic insecurity at the household level. For the dimension on the household risk to an unexpected economic event, I use one indicator and for the dimension on the lack of household economic buffers, I use three indicators (Table 1). This allows me to have a set of indicators that captures vulnerability in different ways. While none of the indicators perfectly captures all aspects of each economic insecurity dimension, taken together, they can be used to identify most of the major sources of stress or risk.

**Table 1:** Dimensions, indicators and cut-offs of the economic insecurity sources

Dimensions	Indicators	Household is vulnerable if…
Household risk to an unexpected economic event	Unexpected economic shocks	the risk of experiencing an unexpected decrease in incomes or an unexpected increase in expenses in the next year is greater than the 70th percentile risk of all households
Lack of buffers to offset potential economic loss	Unprotected employment or non-workers	its workers have not a labour contract and none pay social contributions, or it does not have workers
	Over-indebtedness	the ratio of the monthly payment of the debt to the disposable income of the household is 40% or more
	Asset poverty	assets are insufficient to keep it above the poverty line for 3 months

*Source*: Author’s proposal

All variables are dichotomous

## A Multidimensional Measure of Economic Insecurity

Although the sum of the vulnerabilities presented in Table 2 reveals, with a single measure, the proportion of households that are in a situation of economic insecurity, the index of economic insecurity is not sensitive to changes in the vulnerabilities of the households that are above or below the threshold used. In formal terms, this type of index does not satisfy the properties of dimensional monotonicity. For example, if one were to consider a household economically insecure when it shows two vulnerabilities, the headcount ratio of economically insecure households would not change if a household experiencing three types of vulnerabilities increased to four.

### Using the Adjusted Headcount Ratio to Measure Multidimensional Economic Insecurity

There are several approaches that have been developed to aggregate and summarise information on multidimensional phenomena such as poverty and inequality (Aaberge & Brandolini, 2015). One of the best known is Alkire & Foster’s Multidimensional Poverty Index (MPI) (2011) based on the counting approach (Atkinson, 2003). Alkire and Foster (2011) propose an adjusted headcount ratio as a MPI that is sensitive to changes in the dimensions of the phenomenon that households are facing over time. The empirical strategy is to adapt their approach to the construction of a multidimensional index of economic insecurity.^14^

Economic insecurity also has been measured from a multidimensional perspective following Alkire and Foster (2011) approach in north-western countries. The first time was in the U.S. using cross-sections and panel data from the Survey of Consumer Finances between 1989 and 2009 (Bucks, 2011), and recently in 27 EU countries, using longitudinal EU-SILC data from 2009 to 2016 (Cantó, García-Pérez, & Romaguera-de-la-Cruz, 2020).^15^

The approach that I follow has three parts: (i) the identification of households that are economically insecure, (ii) the aggregation of the different indicators into a scalar value, and (iii) the selection of dimensional weights for each indicator.

#### Identifying Economically Insecure Households

As I described above, I have selected the two dimensions and their indicators which, in the framework are related to household risk to an unexpected economic event and lack of buffers to offset a potential economic loss. Also, I identified economic insecurity for each of the indicators using specific thresholds (see Table 1). The next step is to determine if a household has enough vulnerabilities to be considered economically insecure.

To do this, I build the variable $EI$, which summarizes the total number of economic vulnerability indicators. It is a weighted sum of vulnerabilities in the indicators that define economic insecurity. For a household $i$ it is calculated as follows:

1 $$EI_{i} = \mathop \sum \limits_{j = 1}^{V} w_{j} I_{ij} \quad i = 1, \ldots , n$$

where ${I}_{ij}$ is a variable that takes the value 1 if the household $i$ is vulnerable in the indicator $j$ and 0 otherwise, $V$ is the total number of vulnerabilities analysed, ${w}_{j}$ is the wight assigned to each indicator and $n$ is the total of number of households. The weights are standardised so that their sum equals the total number of indicators, $V$. Therefore, ${EI}_{i}$ will take values between 0 and $V$, where 0 is associated to a household that is not considered to be economic insecurity in any indicators and $V$ is associated to a household $i$ that is considered to be economic vulnerable in all of them.

Once I calculated the $EI$ value for each household, I identify a household as economically insecure from a multidimensional perspective if $EI$ is greater than or equal to the cut-off $k$ (${EI}_{i}\ge k$). And then, the sum of the economically insecure households of $n$ households of the total population is given by ${q}_{EI}$ (${q}_{EI}={\sum }_{i=1}^{n}{I}_{\left\{{EI}_{i}\ge k\right\}}$).

#### Aggregate Economic Insecurity Measures

From an aggregate perspective, I can summarize the information on the economic insecurity of households by one scalar known as ‘adjusted multidimensional headcount ratio’ (${M}_{0}$).^16^ As mentioned the ${M}_{0}$ increases/decreases when the number of economic vulnerabilities increases/decreases, therefore it satisfies the properties of dimensional monotonicity (Alkire & Foster, 2011, p. 481). The ${M}_{0}$ calculates the total weighted sum of economic vulnerabilities divided by the maximum number of vulnerabilities that all households ($nV$) could have experienced. Formally, this expression is:

2 $$M_{0} = \frac{{\mathop \sum \nolimits_{i = 1}^{n} EI_{i} I_{{\left\{ {EI_{i} \ge k} \right\}}} }}{nV}$$

From the perspective of policy analysis the Alkire and Foster (2011) adjusted headcount ratio has two characteristics that make it an appropriate measure. First, it simultaneously measures both the incidence (proportion of economically insecure households) and the intensity of the economic insecurity (number of vulnerabilities affecting it). Second, it can be decomposed by population subgroup (e.g. income decile groups or geographic area) and economic insecurity indicators (e.g. unexpected economic shocks, unprotected employment, over-indebtedness, and asset poverty).

Regarding the former, I can calculate the (${M}_{0}$) using the product of both the incidence ($H$) and the intensity ($A$) of the economic insecurity phenomenon.

3 $$M_{0} = HxA$$

To measure the incidence of economic insecurity in the population, I calculate the ‘multidimensional headcount ratio’ as follow^17^:

4 $$H = \frac{{q_{EI} }}{n}$$

Then I measure the intensity of economic insecurity as the average of the vulnerabilities faced by economic insecure households standardised (${u}_{EI}^{{q}_{EI}}={\sum }_{i=1}^{n}{{EI}_{i}I}_{\left\{{EI}_{i}\ge k\right\}}/{q}_{EI}$) by the total number of indicators of economic vulnerability $V$.

5 $$A = \frac{{u_{EI}^{{q_{EI} }} }}{V}$$

Replacing $H$ and $A$ in Eq. 3, I get Eq. 2 since ${q}_{EI}{u}_{EI}^{{q}_{EI}}={\sum }_{i=1}^{n}{I}_{\left\{{EI}_{i}\ge k\right\}}$

6 $$M_{0} = \frac{{q_{EI} }}{n}\frac{{u_{EI}^{{q_{EI} }} }}{V}$$

Regarding the latter, the ${M}_{0}$ is additively decomposable by population subgroup, and also by vulnerabilities (Alkire & Foster, 2011). Therefore, the ${M}_{0}$ can be expressed as a weighted sum of the adjusted headcount ratios of each of the $S$ subgroups:

$$M_{0} = \mathop \sum \limits_{l = 1}^{S} \frac{{n_{l} }}{n}M_{{0}_{l}}$$

where ${n}_{l}$ is the size and ${{M}_{0}}_{l}$ is the the adjusted headcount ratio of subpopulation $l$.

The ${M}_{0}$ can also be decomposed by vulnerabilities as follows:

$$M_{0} = \mathop \sum \limits_{j = 1}^{V} \frac{{H_{j} }}{V}$$

where ${H}_{j}$ is the proportion of the total number of economically insecure households with elements of vulnerability on dimension $j$.^18^

I assign the weights using a normative approach to define the economic insecurity index.^19^ It uses uniform weights, that is, the index of economic insecurity that has four indicators (treated as dimensions) whose weight (${w}_{j}$) takes the value of 1 for each of them. I call this measure the Multidimensional Economic insecurity index (MEII), which has the following expression:

7 $$MEII_{i} = \left\{ {\begin{array}{*{20}l} {1 if \mathop \sum \limits_{j = 1}^{V} w_{j} I_{ij} \ge k{\text{ where }}w_{j} = 1{\text{ and V}} = { }4} \\ {0 \quad {\text{otherwise}}} \\ \end{array} } \right.$$

## Results

### Economic Insecurity in Chile: An Overview

This section provides a descriptive analysis of the four indicators of economic insecurity to contextualise economic insecurity in Chile between 2007 and 2017. Table 2 shows the behaviour of the insecurity measures constructed with the SHF data. In aggregate, the indicators deliver a broad and clear definition of economic insecurity. When combining all of the years, 8 out of 10 households are classified as economically insecure in at least one of the four measures during the decade studied. Half of the population is classified as economically insecure when considering two or more vulnerabilities. When using a more demanding criterion, that is, three or more vulnerabilities, 13.9 per cent are economically insecure, and only 1.6 per cent of households are in a situation of insecurity in the four indicators.

**Table 2:** Shares of households classified as economically insecure in Chile, 2007–2017

Dimensions and indicators	Headcount ratio: 2007–2017	SHF cross-section survey year				Time trend: 2007–2017
		2007	2011	2014	2017	
*Household risk to an unexpected economic event*						
Unexpected economic shocks	37.9	43.7	37.9	31.5	38.5	−5.3*
*Lack of buffers to offset potential economic loss*						
Unprotected employment or non-workers	30.0	34.0	37.6	23.8	24.5	−9.5**
Over-indebtedness	15.4	15.1	13.5	15.2	17.6	2.6*
Asset poverty	62.8	67.2	72.7	56.2	55.1	−12.1**
*Households by number of vulnerabilities*						
One (any) vulnerability	81.0	84.6	86.8	75.1	77.7	−6.9**
Two or more	49.5	55.4	56.9	40.6	45.1	−10.3**
Three or more	13.9	18.0	16.0	9.9	11.6	−6.4**
Four	1.6	1.9	2.1	1.1	1.3	−0.6*
Number of households		3827	4057	4502	4549	

*Source*: Author’s calculations based on the Chilean Survey of Household Finances (2007, 2011, 2014 and 2017)

**Significant at 5 percent; * significant at 1 percent

The estimated trends are shown in the last column of Table 2. The indicator that measures the risk of households facing an economic shock presents a significant negative tendency during the period analysed, despite the increase from 31.5 per cent to 38.5 per cent between 2014 and 2017. This indicator appears coupled with the changes in national unemployment and GDP growth rates.

**Fig. 1:**
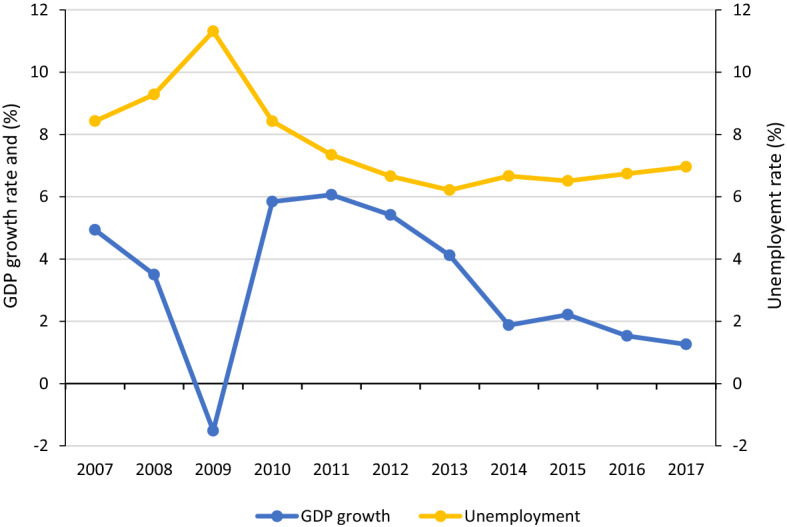
Evolution of economic growth and unemployment in Chile, 2007–2017.* Sources*: For GDP growth, data from OECD Economic Outlook 102 database, and for unemployment data from International Labour Organization, ILOSTAT database

Figure 1 shows that after the economic crisis in 2008, the annual unemployment rate rose to 11.3 per cent in 2009, and then began to decline during the economic expansion period, reaching 6.2 per cent in 2013. Since then the rate of unemployment has slightly increased. Likewise, the economic growth recovered by 2010, reaching similar rates to that before the financial crisis; it then fluctuated at around 5.5 per cent per year until 2013, after which time there was a substantial decline (about 1.7 per cent annually).

Concerning the lack of protection of households to offset an economic loss, significant improvements are observed. The proportion of households with workers without access to social protection mechanisms or non-workers decreased from 34.0 per cent in 2007 to 24.5 per cent in 2017. Likewise, the proportion of households without enough private assets to face an event with negative economic consequences fell from 67.2 per cent in 2007 to 55.1 per cent in 2017. The highest levels of economic insecurity were reached in 2011 when 37.6 per cent of households were either in unprotected jobs or had non-worker members, and 72.7 per cent of households were asset poor. Only the over-indebtedness of households significantly increased during the period studied. In 2017, 17.6 per cent of households showed a high risk of default.

**Fig. 2:**
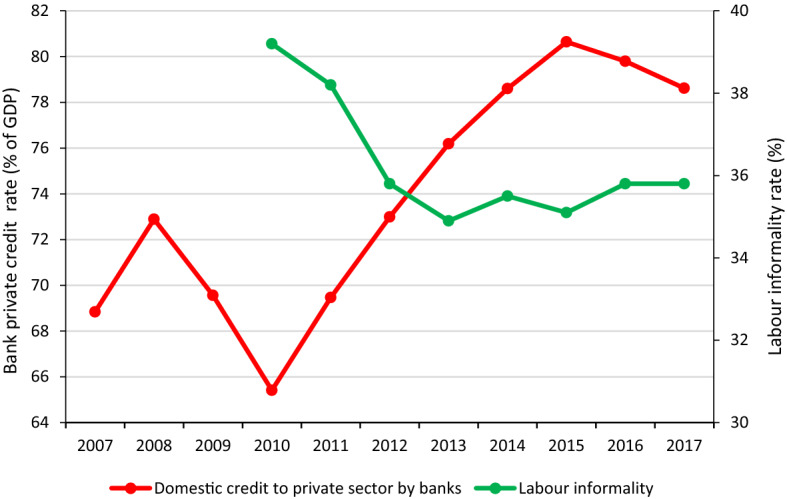
Evolution of bank credit to GDP and labour informality in Chile over time.* Sources*: For labour informality data from New National Employment Survey (known as NENE in Spanish which began to be applied on 2010), and for domestic credit to private sector by banks, data from World Bank, databank

The tendencies of these three measures of household buffers to offset an unexpected economic loss can be somewhat contrasted with macro indicators. For instance, the asset poor households follow the macro changes in the economy and labour market (Fig. 1). In the case of households with unprotected employment, it is not evident that this is related to a decrease in unemployment. This can be associated with either an increase in informal jobs or with an increase in the rate of labour-protected jobs. Figure 2 clarifies this point. Between 2010 and 2013, the proportion of informal work fell from 39.2 per cent to 34.9 per cent in the Chilean labour market. However, in the following years, the informality rate increased slightly, reaching 35.8 per cent in 2017. As to the level of households’ over-indebtedness, this indicator follows the trend of financial resources allocated by domestic money banks. Figure 2 shows that between 2010 and 2017, the bank private credit rate increased by 13.2 per cent, peaking at 80.6 per cent in 2015.

Table 3 shows the association between economic insecurity measures. A third of households with unprotected workers or non-workers are at risk of facing an unexpected economic shock. In the case of the over-indebtedness indicator, 46 per cent of households have a high probability of experiencing an event that generates an economic loss. As to the asset poverty indicator, 44.5 per cent of households in this situation are at risk of having a significant income drop or higher expenses in the near future. It is worth noting that the strongest correlation is between asset poverty and unexpected income shocks at 0.21 (see Table 8 in the appendix). This minimises the problem of double counting, which, as I will discuss in the next section, is one of the critiques to multidimensional approaches.

**Table 3:** The joint distribution between economic insecurity indicators in Chile, 2007–2017

Indicators	Per cent of households in row meeting column criterion (%)			
	Unexpected economic shocks	Uninsured employment	Over-indebtedness	Asset poverty
All households	37.9	30.0	15.4	62.8
Unexpected economic shocks	–	25.9	18.6	73.5
Unprotected employment or non-workers	32.9	–	15.1	70.2
Over-indebtedness	46.0	29.5	–	72.7
Asset poverty	44.5	33.5	17.9	–

*Source*: Author’s calculations based on the Chilean Survey of Household Finances (2007, 2011, 2014 and 2017)

### General Analysis of Economic Insecurity Measures

**Fig. 3:**
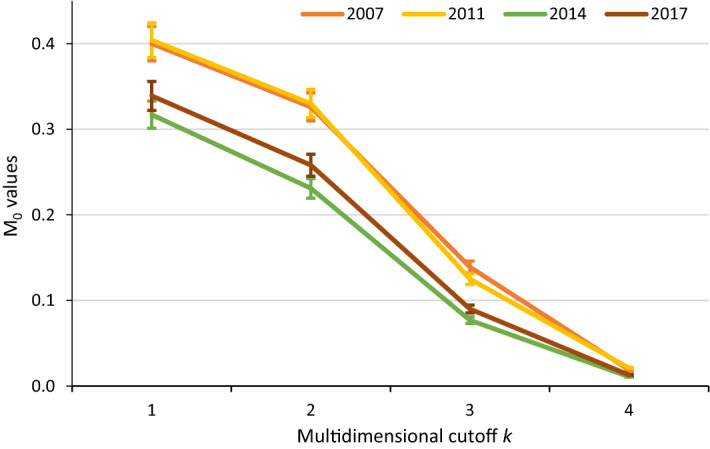
Adjusted multidimensional economic insecurity rate (M_0_) using uniform weights by number of *k* cut-off (Chile, 2007–2017).* Source*: Author’s calculations based on the Chilean Survey of Household Finances (2007, 2011, 2014 and 2017)

The results at the household level of the economic insecurity measures are presented below, and why these results justify using the MEII to understand the economic insecurity in Chile are discussed. Figure 3 shows the aggregate measure (${M}_{0}$) of the MEII for different thresholds ($k$). For the years 2007 and 2011, the confidence intervals overlap for each of the cut-offs, thereby presenting no significant differences in the ${M}_{0}$. For $k$ = 1, the value of ${M}_{0}$ for those 2 years is 0.4, reaching 0.02 when the household experiences the four vulnerabilities at the same time. When analysing the period 2014–2017, the values of ${M}_{0}$ are statistically different when the cut-off corresponds to two vulnerabilities ($k$ = 2). This shows that the economic insecurity behaviour follows a U shape, that is, there is a significant drop in economic insecurity between 2011 and 2014, which is then followed by an increase in the MEII between 2014 and 2017.

To calculate the MEII I use $k$ = 2, a threshold that, as shown in Fig. 3, distinguishes significant changes in the economic insecurity of Chilean households after the Great Recession in 2008/2009. These changes range from an ${M}_{0}$ of 0.330 in 2011 to 0.258 in 2017, where the lowest level of economic insecurity was observed in 2014, with a value of 0.231.

Table 4 shows the aggregate measures for the MEII ($H$, $I$, and ${M}_{0}$) for k = 2. The changes in the adjusted multidimensional economic insecurity rate (${M}_{0}$) are explained more by variations in the incidence ($H$) (over 12 per cent between 2007 and 2017), than by changes in the intensity ($I$) of the vulnerabilities (less than 3 per cent for the same period). However, although the level of economic insecurity taken in 2007 and 2011 was high (more than half of the population) and did not change, its evolution in the subsequent years shows U-shaped behaviour where a significant fall in $H$ between 2011 and 2014 follows an increase between 2014 and 2017. This result raises the question of what the contribution of each of the indicators is to ${M}_{0}$, and how this contribution changed over the decade studied.

**Table 4:** Measurements of economic insecurity in Chile, 2007–2017

Index defined by weights and threshold	Year	H (incidence of economic insecurity)	Std. Err	A (intensity of economic insecurity)	Std. Err	M_0_ (adjusted multidimensional economic insecurity rate)	Std. Err
MEII (Multidimensional Economic insecurity index) Four dimensions, uniform weights and k = 2	2007	0.554	0.012	0.590	0.005	0.326	0.007
	2011	0.569	0.009	0.579	0.004	0.330	0.006
	2014	0.406	0.012	0.568	0.005	0.231	0.007
	2017	0.451	0.010	0.571	0.004	0.258	0.006
	∆ 2007–2017	−0.103	**	−0.019	*	−0.068	**

*Source*: Author’s calculations based on the Chilean Survey of Household Finances (2007, 2011, 2014 and 2017). ** significant at 5 percent; * significant at 1 percent

### Disaggregated Analysis by Dimensions, Income Decile Groups and Family Types

**Fig. 4:**
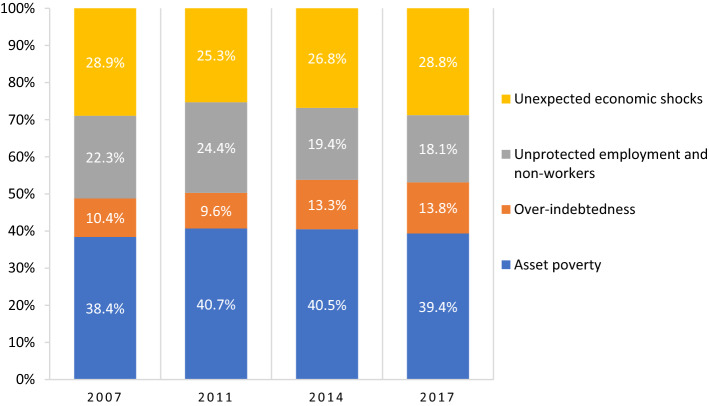
Evolution of the relative composition of MEII (M_0_) in Chile, 2007–2017.* Source*: Author’s calculations based on the Chilean Survey of Household Finances (2007, 2011, 2014 and 2017). Notes: The MEII (Multidimensional Economic insecurity index) uses uniform weights and k = 2

Figure 4 illustrates the evolution of the composition of ${M}_{0}$. In the four measurements obtained between 2007/2017, asset poverty is the dimension that contributes the most to economic insecurity, with an average of 40 per cent. The second most important component of the ${M}_{0}$ for all households is unexpected economic shocks, with an average of 28 per cent. In third place is unprotected employment, with an average of 21 per cent. Finally, the component with the lowest contribution to the aggregated measure of the MEII is over-indebtedness, at 11 per cent. Yet, it is worth noting that this dimension is the only one out of the four dimensions considered that increased its contribution over the decade (from 10.4 per cent in 2007 to 13.8 per cent in 2017). The changes in the compositions of the other three dimensions become more apparent from 2011 onwards; the contribution of economic shocks increases, and the lack of buffers (asset poverty and unprotected employment) decreases. In 2017 the relative ${M}_{0}$ composition of these three dimensions was 28.8, 39.4 and 18.1 per cent respectively.

**Fig. 5:**
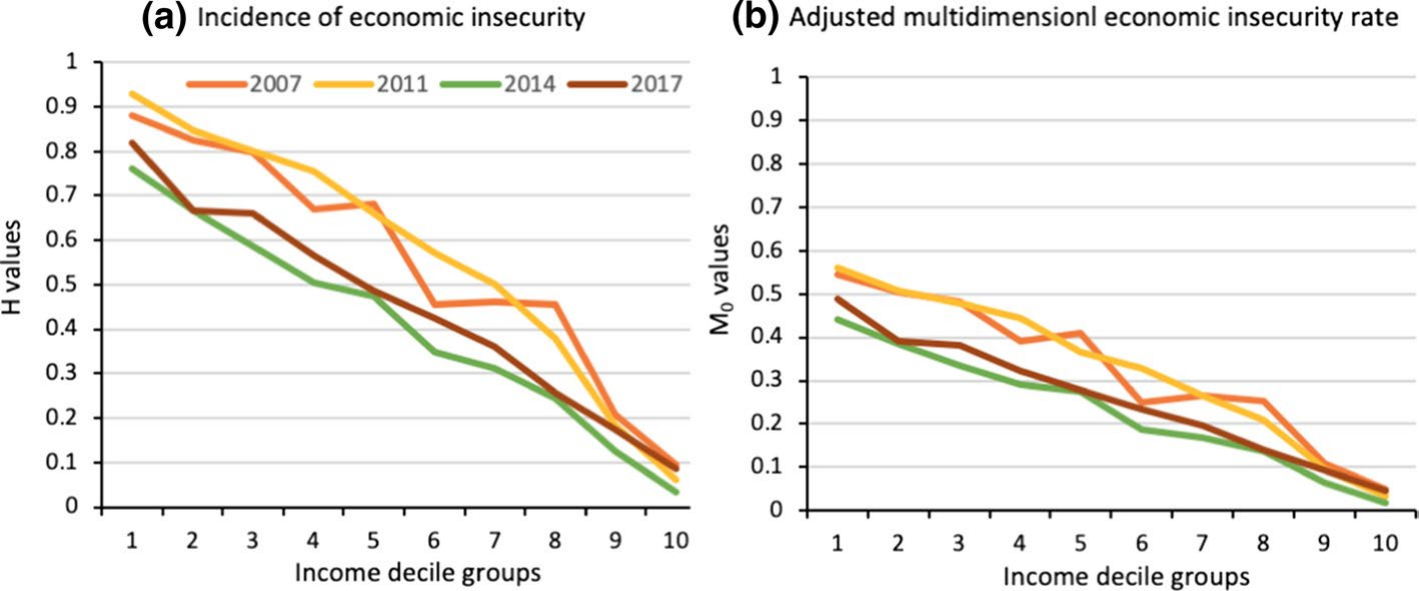
Aggregate measures of MEII by income decile groups in Chile, 2007–2017.* Source*: Author’s calculations based on the Chilean Survey of Household Finances (2007, 2011, 2014 and 2017)

Figure 5 shows the aggregate MEII measures for the years 2007, 2011, 2014, 2017 by income decile group. The results show two relevant phenomena. First, economic insecurity affects the whole population. As expected, the economic insecurity is much higher in the lower part of the income distribution and decreases when moving to the higher income deciles. Panel A in Fig. 5 shows that during the years 2007 and 2011, the average incidence of the MEII was around 80 per cent for the first two income decile groups, while in the two highest income decile groups (9 and 10) was 12 per cent. The proportion of economic insecurity in the upper part of the distribution contrasts with the results from a recent study that has carried out a similar analysis using a multidimensional index developed by Romaguera de la Cruz (2020). This author found that in Spain, France and Sweden, the ${M}_{0}$ for deciles 9 and 10 was less than 1 per cent. Although this comparison is not strictly accurate since the period analysed was between 2009/2015 and the index was not built with the same indicators, it allows for emphasising the fact that economic insecurity in Chile is not bounded to the lower income groups.

This result is in line with evidence collected through qualitative studies in Chile that show a pervasive fear experienced by the middle-upper class of not remaining in their social position due to economic problems (Araujo & Martuccelli, 2011). In a context of a weak welfare state offering low-quality services, the upper-middle class in Chile has turned to private providers of services such as education and health (Méndez & Gayo, 2019). Further, Pérez-Roa and Ayala (2020) find that young professionals, who recently joined the high-income ranks, tend to be economically fragile due to their deep indebtedness (particularly among those who received post-secondary loans).

This result is particularly interesting when comparing the concept of economic insecurity with that of vulnerability to poverty (risk of falling into poverty). Poverty vulnerability analyses indicate that in Chile, only the highest income decile groups (9 and 10) have a near zero risk of falling into poverty. This means that a low risk in terms of vulnerability to poverty does not exempt households or individuals from the risk of curtailing their well-being, a risk that is associated with significant stress at the household level.

The second phenomenon that the results show is that although in Chile the levels of economic insecurity are high, these decreased significantly between 2011 and 2014. Figure 5 shows that this reduction is reflected throughout the first eight income decile groups in both aggregated measures ($H$ and ${M}_{0}$). The highest income decile groups (9 and 10) show no significant changes. As noted, this decrease in economic insecurity is coupled with good macroeconomic performance between those years: economic growth of 4.4 per cent on average and a decrease in informal work of almost 5 per cent.

Taking advantage of the decomposition properties of one of the MEII’s aggregated measures, I analyse the contribution of the dimensions to economic insecurity according to households’ position in the distribution of income. And, more specifically, I analyse whether the composition of the four dimensions in the index differs between the extremes of the income distribution. Table 5 shows the adjusted multidimensional economic insecurity rate (${M}_{0}$) for the year 2017 by income decile group.

**Table 5:** Relative contribution to M_0_ by income decile group in Chile, 2017

Income decile groups	M_0_ (adjusted multidimensional economic insecurity rate)	Relative contribution to M_0_			
		Unexpected economic shocks	Unprotected employment or non-workers	Over-indebtedness	Asset poverty
1	0.490	0.178	0.346	0.078	0.397
2	0.390	0.257	0.253	0.097	0.393
3	0.383	0.304	0.169	0.131	0.396
4	0.322	0.319	0.143	0.137	0.401
5	0.278	0.366	0.097	0.157	0.380
6	0.235	0.364	0.107	0.158	0.371
7	0.198	0.355	0.060	0.174	0.412
8	0.139	0.351	0.076	0.164	0.409
9	0.092	0.197	0.057	0.338	0.408
10	0.046	0.199	0.156	0.312	0.333

*Source*: Author’s calculations based on the Chilean Survey of Household Finances 2017

For the year 2017, the contribution of unexpected economic shocks is higher between deciles 3 and 8 than at the extremes of the income distribution. In the case of asset poverty, the contribution is relatively constant. It does not seem to be related to income decile, except in the highest income decile group (10), where the contribution falls to 33 per cent. The indicator unprotected employment or non-workers in the household is important in the lower part of the income distribution, and its contribution falls in the highest deciles. Conversely, over-indebtedness is more relevant for households at the top of the distribution, which reveals that over-indebtedness, falling revenues or increased expenditure are sources of greater stress among households with a higher income in Chile.

The breakdown of the MEII aggregate measures into subgroups of the population also makes it possible to identify the types of families with the highest levels of economic insecurity. From the perspective of public policy design, this information is relevant because it allows for identifying where and how to focus public resources to reduce household stress due to economic vulnerabilities, thus complementing other welfare measures that are traditionally used in the targeting of social policies.

**Fig. 6:**
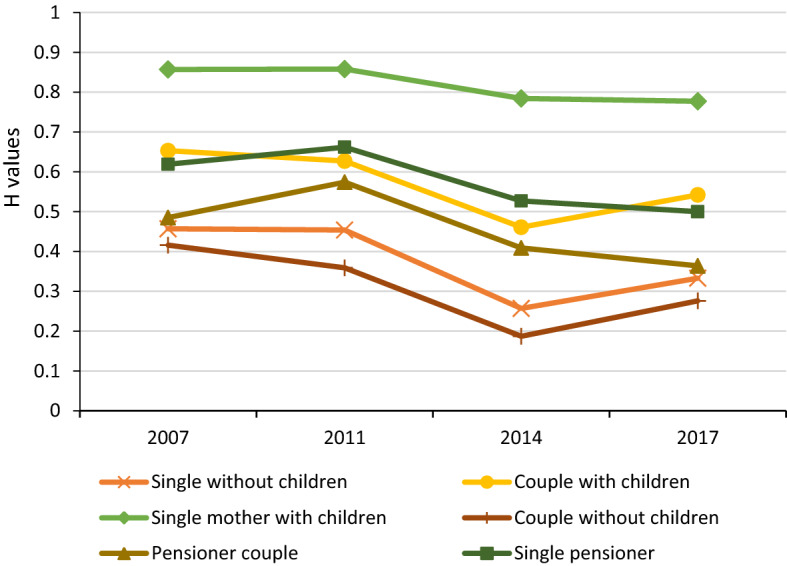
Incidence of economic insecurity ($H$) by family type in Chile, 2007–2017.* Source*: Author’s calculations based on the Chilean Survey of Household Finances (2007, 2011, 2014 and 2017)

Figure 6 shows the incidence of economic insecurity in Chile between 2007 and 2017, broken down by population subgroup. The three groups with the highest rate of economic insecurity are households composed by (i) a single mother with children; (ii) couple with children, and (iii) a single pensioner. Around 8 out of 10 single mother with children households experienced economic insecurity during the decade analysed. The other two subgroups show rates close to 65 per cent in 2007. Although by 2017 these rates had declined to around 52 per cent. The high economic insecurity of these three types of families correlates with other welfare deprivation measures such as vulnerability to income poverty (López-Calva & Ortiz-Juarez, 2014). In this way, the application of the MEII informs policymakers that more than half of these households have been under economic stress during the last decade in Chile.

### Understanding the Determinants of the Economic Insecurity in Chile

The last section of the results analyses the relationship between economic insecurity and some significant households’ characteristics variables. I use a probit model to estimate the probability of a household being economically unsafe. The dependent variable is the definition of the MEII for a cut-off of two vulnerabilities. The multivariate model was applied to pooled data from SHF household samples for the years 2007, 2011, 2014 and 2017. Specifically, the interest is in identifying the average marginal effect (AME) that each of the socioeconomic characteristics of the household has on economic insecurity for the studied period. Table 6 shows these estimates.

**Table 6:** Average marginal effects on probability of a household being economic insecure for significant variables

Variables	Pooled data	
	AME	Std. Dev
*Household head characteristics*		
Female	0.077***	(0.017)
Age: Ref.45 to 54 years		
Under 35 years	−0.024	(0.022)
35 to 44 years	−0.010	(0.021)
55 to 64 years	−0.025	(0.024)
65 years and more	0.049	(0.051)
Education: Ref. Secondary school		
Primary school	0.079***	(0.020)
University degree	−0.260***	(0.014)
*Household characteristics*		
Household type: Ref. Couple with children		
Single without children	0.016	(0.023)
Single mother with children	0.118***	(0.029)
Couple without children	0.273***	(0.040)
Pensioner couple	−0.081	(0.050)
Single pensioner	−0.093*	(0.050)
Number of children < 15	0.031**	(0.014)
Number of workers	−0.110***	(0.007)
Housing: Ref. Own housing (no mortgage)		
Own housing, mortgage	0.179***	(0.016)
Rent	0.012	(0.021)

*Source*: Author’s calculations based on the Chilean Survey of Household Finances (2007, 2011, 2014 and 2017)

I present average marginal effects for probit estimations in which the dependent variable is the Multidimensional Economic Insecurity Index (MEII)

***Significant at 10 percent; ** significant at 5 percent; * significant at 1 percent

**Table 7:** Average marginal effects on the probability of a household facing a large drop in income or a sharp increase in its expenses for significant variables

Variables	Pooled data: 2007–2011 to 2014–2017	
	AME	Std. Dev
*Household head characteristics*		
Female	0.020**	(0.008)
Age (years)	0.005***	(0.001)
Age^2^ (years)	−0.001**	(0.001)
Education: Ref. Secondary school		
Primary school	0.021**	(0.011)
University degree	0.002	(0.013)
Labour status: Ref. Unoccupied		
Formal employed	0.004	(0.011)
Informal employed	0.036***	(0.012)
*Household characteristics*		
Household type: Ref. Couple without children		
Single without children	−0.033**	(0.015)
Couple with children	0.053***	(0.015)
Single mother with children	−0.020	(0.015)
Pensioner couple	−0.024	(0.020)
Single pensioner	−0.069***	(0.022)
Number of children < 15	0.026***	(0.007)
Number of workers	0.015***	(0.005)
Housing: Ref. Own housing (no mortgage)		
Rent	0.043***	(0.009)
Own housing, mortgage	0.025**	(0.011)
Income: Ref. Decile 6–8 income group		
Decile 1–5 income group	0.030***	(0.010)
Decile 9–10 income group	−0.078***	(0.010)
Year: Ref. 2017		
2007	0.026**	(0.011)
2011	0.002	(0.010)
2014	−0.085***	(0.010)

*Source*: Author’s calculations based on the Chilean Survey of Household Finances (2007, 2011, 2014 and 2017)

I present average marginal effects for probit estimations in which the dependent variable is a large drop in household income or a sharp increase in its expenses

***Significance at 10 percent; ** significance at 5 percent; * significance at 1 percent

**Table 8:** Correlation between economic insecurity indicators in Chile, 2007–2017

Indicators	Per cent of households in row distributed in each indicator (%)			
	Unexpected economic shocks	Uninsured employment	Over-indebtedness	Asset poverty
Unexpected economic shocks	1			
Unprotected employment or non-workers	0.047	1		
Over-indebtedness	0.144	-0.003	1	
Asset poverty	0.213	0.141	0.096	1

*Source*: Author’s calculations based on the Chilean Survey of Household Finances (2007, 2011, 2014 and 2017)

Regarding the features of heads of households, i.e. gender, age and education, the results show first, that households headed by women are more vulnerable than households headed by men (7.7 per cent). An explanation of this result could be the gender inequalities that the Chilean labour market exhibits (participation, stability and wages). Second, the age of the head of the household was not a significant variable. This result shows that the economic insecurity throughout the decade was transverse to the life cycle of households. Finally, it is worth noting that households with heads of households that have a university degree have a significantly reduced risk of being economically vulnerable. The AME for heads of households with a university degree was 26.0 percentage points.^20^

As to the variables related to households' characteristics, i.e. type of family, number of children, number of members working, the results in Table 6 show that two types of households have a higher risk of being economically insecure compared to households with couples without children. In the case of single mother with children, the risk rises by 27.3 percentage points, while for couples with children, it increases by 11.8 percentage points. These results are aligned with those presented in Fig. 6, providing significant evidence of the need to direct support through tailored policies to these types of families to alleviate the stress and anxiety they experience. It is important to mention that during the last decade, Chile has been increasing the cash transfer through its family benefit programs, reaching 0.7 per cent of GDP in 2015 (Tromben & Podestá, 2019, p. 59). However, this percentage is still below the average of 1.2 percent from OECD countries.

Second, regarding the number of children in the household, an additional child increases the probability of a household being economically insecure by 3.1 percentage points. Third, the number of workers in the household has a reverse effect and is significant. When a member of the household gets a job the probability of the household being economically insecure decreases by 11.0 percentage points. Finally, renting, as opposed to owning, increases the probability of being economically vulnerable by 17.9 percentage points. Since housing was not included as a measure of asset poverty, this result does not have a mechanical explanation but rather directly relates this characteristic of the household to the level of insecurity that it experiences.

## Conclusions

This paper has studied both the nature and evolution of economic insecurity in Chile over the last 10 years (from 2007 to 2017). A Multidimensional Economic Insecurity Index (MEII) has been constructed to carry out this analysis, combining four sources of economic insecurity that causes stress and anxiety: unexpected economic shocks, unprotected employment, over-indebtedness, and asset poverty. In this way, the MEII offers a measure at the household level that directly relates economic uncertainty to stress due to the lack of both social protection and buffers to face unexpected economic shocks.

The MEII incorporates sources of stress and anxiety that are characteristic of households located at the two ends of the income distribution in middle-income countries. This is the case for the unprotected jobs at the bottom of the distribution, and the over-indebtedness at the top. In this way, the MEII becomes a more versatile and useful tool for the diagnosis and design of social policies for the reality of countries such as Chile and others that are similar in the Latin American region. Furthermore, using a multidimensional approach to construct the MEII not only allows me to analyse the incidence and intensity of economic insecurity but also to decompose the index by dimension or subpopulation.

After selecting the appropriate vulnerability cut-off to the MEII, a cut-off of two vulnerabilities and four indicators with uniform weights is proposed to analyse the level and intensity of economic insecurity in Chile. Applying this measure to the data from the Household Financial Survey shows that during the decade studied, economic insecurity, on average, affected almost 50 per cent of urban households in Chile, with an intensity of 2.3 out of 4 indicators. By considering both incidence and intensity, an adjusted rate of average economic insecurity of 0.286 is obtained. Although in the period of the economic crisis the level of insecurity did not change (measures taken in 2007 and 2011), its evolution in the subsequent years shows U-shaped behaviour where a significant fall in economic insecurity between 2011 and 2014 is followed by an increase between 2014 and 2017. This result shows a negative correlation with the country’s economic cycle. Other macroeconomic indicators also correlate with some of the indicators that make up the MEII, for example, the reduction of the levels of labour informality with the unprotected unemployment indicator, and the constant increase in the bank private credit rate with the over-indebtedness indicator.

When considering the entire population, asset poverty is the indicator that contributes the most to economic insecurity. The other indicators follow in this order: unexpected economic shocks, unprotected employment, and over-indebtedness. Although insecurity is present throughout the income distribution, the composition of the four indicators varies according to the position of the household in the income deciles. Thus, although the asset-poverty contribution is similar throughout the income distribution, unprotected employment is more relevant in the lower deciles, while unexpected economic shocks and over-indebtedness make a more significant contribution in the higher deciles.

The main determinants of economic insecurity are households headed by women who have children. Also, heads of households with low educational levels who work without a contract increase the household’s risk of being affected by economic insecurity. The number of workers in the household is the most critical determinant to predict their economic insecurity. These results are similar to studies that have used other economic welfare measures such as vulnerability to poverty. This allows for relating these forms of socioeconomic disadvantage to exposure to economic stress. In this way, one could argue that policies that seek to reduce the economic risk in the poorest households fulfil several desirable objectives simultaneously.

The most significant difference between these welfare measures is that economic insecurity affects the entire income distribution, while the other measures do not provide relevant information on the highest deciles. The high economic insecurity experienced by all income groups finds an explanation in two critical and intertwined conditions: firstly, the low level of income and wealth collected through household surveys, even of those in decile groups 9 and 10, which are not enough to protect individuals from the stress of future economic shocks; and secondly, the weak social protection system, which is incapable of working as a buffer to offset households’ economic insecurity. It is worth noting that in 2015 the OECD ranked Chile as having the greatest economic vulnerability among its members, for almost 8 out of every 10 Chileans did not have liquid financial wealth to face a sudden adverse economic shock. In that same year a new reform was made to the unemployment insurance system based on individual savings to increase insurance coverage for a greater proportion of the unemployed.

By identifying the groups of households most affected by economic insecurity and its trend in recent years, the application of the MEII in countries such as Chile provides relevant information to monitor, evaluate and improve social safety nets together with labour market regulations. Although this welfare measure has been criticised for not considering the fact that the perception of economic vulnerability varies among households, it is important to acknowledge that the contexts in which households decide to avoid or increase their economic risks are determined and informed by the support scheme offered through social policies. The question that arises is, what is the base level of hazard that as a society we want to have? In the case of Chile, to a certain extent, the state shares with people the financial risk of hazards such as unemployment or illness, through programmes such as unemployment insurance or public health insurance. Households decide how to cope with the additional costs of an illness or unemployment taking into consideration information about programme benefits (if eligible) and their own resources. However, regardless of the level of risk aversion on the part of the household, social policies should be able to effectively address economically insecure households, generating a more complete social welfare system than the current one.

## Appendices

### Using a Normative Approach to Define the Weighting Structure

Using an appropriate weighting scheme for any compound index is crucial.Weights have critical importance in the construction of a measure of wellbeing because they determine the trade-off between the dimensions and/or indicators, which can significantly affect the conclusions derived from the index (Decancq & Lugo, 2013; Ravallion, 2012). The weights given to the different sources of stress that the household has due to economic vulnerabilities are a determining factor in the definition of the index I propose.^21^ There are several approaches to setting weights, which can be grouped into two types. The first are the data-driven approaches, which let the data ‘speak for themselves’ and depend solely on the distribution that the data being analysed provides. That is, data-driven weights are based on neither theoretical criteria nor value judgements regarding what the trade-offs should be between the dimensions and indicators. The second are the normative approaches, which define the weights based only on value judgements or conceptual frameworks of the dimensions of the phenomenon studied rather than the information that the distribution of the data matrix can provide (Table 7).

There are two reasons for not using data-driven weighting strategies such as the principal component analysis (used by Rhode et al. (2015) to build the multidimensional index of economic insecurity in Australia) or frequency-based weights (used by Romaguera de la Cruz (2020) in her index for Spain, France and Sweden). First, as mentioned in the previous section, the indicators that I use for Chile do not have a high correlation with each other, minimising the problem of double counting, which can capture the same latent dimension for two highly correlated indicators. The use of techniques based on principal component analysis has some drawbacks such as the difficulty in interpreting the combination between the indicators of the index, and in assigning a low weight to the dimensions that show a weak correlation, relying on mechanic justifications rather than theoretical ones (Decancq & Lugo, 2013).

Second, in countries like Chile that show a high percentage of economic vulnerability in all the indicators, using frequency-based weights has no justification. The reason for using frequency-based weights is that households attach greater importance to vulnerabilities that do not affect most households. Besides, there are situations in which one dimension may have a significant impact on the population, but this does not mean the others with a lower impact are less important. For example, Brandolini (2007) found this type of inconsistency between dimensions such as health and education when using frequency-based weights to calculate a well-being index in Italy.

Within the normative approach, the equal weighting is the most commonly used approach to build multidimensional indices of well-being (e.g. Human Development Index (UNDP, 2018)). It is recommended when the dimensions used in the index are considered equally important or when dimensions that do not overlap are included. In my framework, the dimensions contain both characteristics. First, the vulnerability indicators are not highly correlated (see Table 8 in the appendix). Second, each dimension has an adverse effect on the well-being of a household, and, to the best of my knowledge, there are no studies that have determined that an economic vulnerability (e.g., over-indebtedness) causes more stress or anxiety in the household members than another (e.g., unprotected employment). Therefore, by applying the same weight to each indicator of economic insecurity, I can treat them in the same way.

## Footnotes

1 For reviews, see Osberg (2018) and Hacker (2018).

2 For a detailed review of synthetic indicators, see Maggino (2017).

3 The proposal of a multidimensional index to measure economic insecurity is not new. Bucks (2011) measured economic insecurity in the United States using twelve household-level measures of (i) vulnerability to health, employment, or income shocks, (ii) adequacy of household savings and income, and (iii) borrowing constraints. His index is based on the methodology proposed by Alkire & Foster (2011) for measuring multidimensional poverty. Unlike Hacker et al. (2014), Bucks (2011) did not find a significant increase in economic insecurity except during the Great Recession (2007–2009).

4 In these countries vulnerability to poverty is the concept that is most often examined. It has helped in thinking about how to protect people from the risk of a decline in their well-being (López-Calva & Ortiz-Juarez, 2014).

5 The SHF methodological documents, reports and databases can be accessed through the following link: https://www.bcentral.cl/financiera-de-hogares.

6 These characteristics have made it possible to include the Chilean SHF in the OECD Wealth Distribution Database, which has been used for comparative studies on households’ wealth inequality (Balestra & Tonkin, 2018; Murtin & d’Ercole, 2015; OECD, 2013).

7 The use of equivalence scales for the estimation of household income in Chile began in 2013. The value of the equivalence elasticity was defined by an Expert Advisory Committee to update the poverty line (Ministerio Desarrollo Social, 2015).

8 In 2007 the SHF did not include this question. Therefore, I used as a proxy the SHF 2007 question if household had expenses larger than its income in the last year.

9 Both household head characteristics and household characteristics at $t$, are used as covariables, including gender, age, labour status, educational level, type of households, number of children, number of workers, housing and household income. See the model on Table 7, in the appendices.

10 Bucks (2011), Rohde et al.(2015) and Romaguera de la Cruz (2020) operationalise the income insecure dimension as a binary indicator of whether households experienced a large income drop in the last year, not the risk of facing it. By doing this, in addition to having an ex-post measure instead of a looking-forward measure, they cannot classify as vulnerable households that have not experienced such an economic shock in the previous year.

11 The financial burden corresponds to the monthly repayments for a debt hold by the household.

12 The consumer debt does not require collateral and is known as unsecured debt.

13 The OECD has used this definition of asset poverty to compare the levels of economic vulnerability of member countries (Balestra & Tonkin, 2018). Economically vulnerable households are those that are not income poor but asset poor. The wealth concept used is liquid financial wealth (e.g., bank accounts and other financial assets) because it can be easily monetised, and the period is 3 months. The income poor are those with an equivalised income below 50 per cent of the median income (OECD, 2017, p. 89).

14 It is worth mentioning that Alkire & Foster (2011) MPI has been calculated in 104 countries to identify multiple deprivations at the household level (Alkire & Santos, 2014), and their adjusted headcount ratio has been used in other multidimensional concepts such as job quality. For instance, García-Pérez et al., (2017a, 2017b) for Spain and Sehnbruch et al. (2020) for nine Latin American countries.

15 Cantó et al. (2020) research is based on the Romaguera de la Cruz (2020) work who built an economic insecurity index using an adaptation of Alkire & Foster’s (2011) model proposed by García-Pérez et al., (2017a, b).

16 Alkire and Foster (2011) also propose measures of intensity (${M}_{1}$) and inequality (${M}_{2}$) that are not used in this proposal because the indicators of economic insecurity used are dichotomous, and cardinal data is required for their calculation.

17 The application of this measure, considering that each of the four indicators of economic vulnerability has equal weight, can be seen in the last lines of Table 2.

18 The $H$, $A$ and ${M}_{0}$ estimates were computed in Stata (Release 15.0, Stata Corporation) using the *mpi* command (Pacifico & Poege, 2017).

19 In the Appendices, I explain why I use a normative approach to define the weighting structure of the economic insecurity index that I propose.

20 In other words, in average the probability of these households is around 25 per cent higher than of head of households that only completed secondary school.
